# *SLC2A1* variants cause late-onset epilepsy and the genetic-dependent stage feature: For the China Epilepsy Gene 1.0 Project

**DOI:** 10.1186/s42494-024-00177-0

**Published:** 2024-11-07

**Authors:** Dongming Zhang, Jing Guo, Zisheng Lin, Hongjun Yan, Kai Peng, Linxia Fei, Qiongxiang Zhai, Dongfang Zou, Jiayi Zhong, Yan Ding, Hong Ye, Pengyu Wang, Jie Wang, Sheng Luo, Bingmei Li, Bin Li, Weiping Liao

**Affiliations:** 1 Department of Neurology, Institute of Neuroscience, Key Laboratory of Neurogenetics and Channelopathies of Guangdong Province and the Ministry of Education of China, The Second Affiliated Hospital, Guangzhou Medical University, Guangzhou, 510260 China; 2 Epilepsy Center, Guangdong Sanjiu Brain Hospital, Guangzhou, 510520 China; 3 Department of Pediatrics, Guangdong General Hospital, Guangdong Academy of Medical Sciences, Guangzhou, 510080 China; 4 Epilepsy Center and Department of Neurology, Shenzhen Children’s Hospital, Shenzhen, 518038 China; 5 Department of Neuroelectrophysiology, Guangzhou Women and Children’s Medical Center, Guangzhou Medical University, Guangzhou, 510623 China; 6 Department of Neurology, The First Affiliated Hospital of Jinan University, Clinical Neuroscience Institute of Jinan University, Guangzhou, 510630 China; 7 Epilepsy Center of Foshan First Hospital, Foshan, 528000 China

**Keywords:** *SLC2A1* gene, Glucose transporter type 1 deficiency syndrome, Mild epilepsy, Genetic dependent stage, Seizure-onset age

## Abstract

**Background:**

The *SLC2A1* gene plays a vital role in brain energy metabolism. *SLC2A1* variants have been reported to be associated with early-onset refractory seizures. This study aims to explore the association between the *SLC2A1* gene and late-onset epilepsy.

**Methods:**

Trios-based whole-exome sequencing was performed on patients with epilepsy without acquired etiologies. The pathogenicity of the variants was assessed according to the American College of Medical Genetics and Genomics (ACMG) guidelines.

**Results:**

A total of 14 heterozygous *SLC2A1* variants were identified in 16 unrelated families. The variants were evaluated as “pathogenic” or “likely pathogenic” according to the ACMG guidelines. Ten cases (62.5%) presented with infantile onset seizures and developmental delay/intellectual disability and were diagnosed with developmental and epileptic encephalopathy (DEE). The other six cases (37.5%) exhibited late-onset seizures and normal development. They were diagnosed with idiopathic partial epilepsy (*n* = 2) or idiopathic generalized epilepsy (*n* = 4). Further analysis showed that DEE-associated variants tended to cluster in the transmembrane region, whereas the mild epilepsy-associated variants tended to locate in regions outside the transmembrane region, suggesting a potential molecular sub-regional effect. A total of 15 cases had delayed diagnosis, with the longest delay being 22 years. The *SLC2A1* expression stage, which is expressed at relatively high level throughout the whole life span, from the embryonic to adult stages with two peaks at approximately four and 14 years, is generally consistent with the seizure onset age. In addition, patients with early-onset age had variants that were potentially associated with severe damage, suggesting a potential correlation between the age of disease onset and the damaging effects of the variants.

**Conclusions:**

*SLC2A1* variants are associated with late-onset epilepsy, which is consistent with the genetic-dependent stage feature of *SLC2A1*. Early genetic diagnosis is important for treatment of patients with *SLC2A1* variants.

## Background

The *SLC2A1* gene (OMIM* 138140), which is located on chromosome 1p34.2 and encodes glucose transporter type 1 (GLUT1), is highly expressed in the human blood-brain barrier [1]. GLUT1 is the most important energy carrier of the brain and plays vital roles in brain energy metabolism [2, 3]. Homozygous knockout of *Slc2a1* in mice results in embryonic lethality, whereas heterozygotes exhibit spontaneous seizures, microencephaly, impaired motor performance, hypoglycorrhachia, and reduced brain glucose uptake, suggesting an essential role of *SLC2A1* in neurodevelopment [4].

In humans, variants of *SLC2A1* are associated with glucose transporter type 1 deficiency syndrome (GLUT1DS; OMIM# 606777). GLUT1DS is characterized by delayed neurologic development, acquired microcephaly, motor incoordination, and spasticity [5, 6]. Early infantile refractory seizures are a common feature of GLUT1DS [7]. However, the association between the *SLC2A1* gene and late-onset epilepsy remains elusive.

In this study, we performed trio-based whole-exome sequencing (WES) in a cohort of patients without acquired causes. We identified 14 variants in 16 unrelated cases, including six with late-onset epilepsy. Further analysis suggested that *SLC2A1* is expressed through the embryonic and the adult stages, explaining the onset age of the patients.

## Methods

### Patients

Patients with epilepsy without acquired causes were recruited from the Second Affiliated Hospital of Guangzhou Medical University, Guangdong Sanjiu Brain Hospital, Shenzhen Children’s Hospital, the First Affiliated Hospital of Jinan University, Guangdong General Hospital, and Foshan First Hospital, through the China Epilepsy Project 1.0 platform (www.epg1.cn). Clinical information of the patients was collected from them or their families, including current age, sex, age of seizure onset, seizure type and frequency, anti-seizure medications, growth and development, neurological physical examination, long-term (24-h) video electroencephalogram, and brain magnetic resonance imaging (MRI). The diagnosis of epileptic seizures and epilepsy syndromes was made in accordance with the criteria established by the Commission on Classification and Terminology of the International League Against Epilepsy (1989, 2001, 2010, 2017, and 2022). Patients with acquired epilepsy were excluded.

This study was approved by the Ethics Committee of The Second Affiliated Hospital of Guangzhou Medical University, and written informed consent was obtained from the individuals or legal guardians of the children.

### WES and genetic analysis

Peripheral blood was obtained from the probands and their biological parents (trios), and genomic DNA was subsequently extracted from the blood using the FlexiGene DNA Kit (Qiagen, Hilden, Germany). WES was conducted utilizing the NextSeq2000 sequencing instrument (Illumina, San Diego, CA) in accordance with previously established standard procedures [8–10]. The sequencing data were generated by massively parallel sequencing with > 100 times average depth and > 98% coverage of the capture regions, and the high-quality reads were mapped to the Genome Reference Consortium Human Genome build 37 (GRCh37) by Burrows-Wheeler Alignment (BWA).

A case-by-case analytical approach was used to identify candidate causative variants in each trio [11]. Common variants with a minor allele frequency (MAF) of ≥ 0.005 in the gnomAD database were firstly filtered out. Then, potential disease-causing variants, including nonsense, frameshift, canonical splicing, initiation codon, in-frame, and missense variants predicted to be damaging were chosen for further analysis. Variants were categorized and assessed using the five distinct patterns: (1) epilepsy-associated; (2) dominant or de novo variants; (3) autosomal recessive inheritance (including compound heterozygous and homozygous variants); (4) X-linked inheritance; and (5) co-segregation analysis if available. The variants included in this study met the following standards: (1) the MAF for heterozygous de novo/co-segregated variants is absent in the control populations in gnomAD; (2) for compound heterozygous variants, the product of multiplying the frequencies of two alleles in gnomAD is < 1 × 10^–6^; and (3) homozygotes are not present in the control populations in gnomAD. In this cohort, *SLC2A1* was identified as a candidate gene with recurrent de novo and co-segregation variants. Sanger sequencing was used to validate the candidate pathogenic variants. All the *SLC2A1* variants identified in this study were annotated to NM_006516.

### Temporal expression profile of *SLC2A1* in the brain

The human RNA-seq data in multiple brain areas from 8 postconceptional weeks to 40 years was retrieved from the BrainSpan database (www.brainspan.org). The RNA expression was normalized to the RPKM (reads per kilobase million) value. To interpret the expression pattern of *SLC2A1*, the temporal expression curve was modeled using a third-order polynomial regression analysis via the least squares fitting implemented in GraphPad Prism 9.

## Results

### Identification of *SLC2A1* variants

**Fig. 1:**
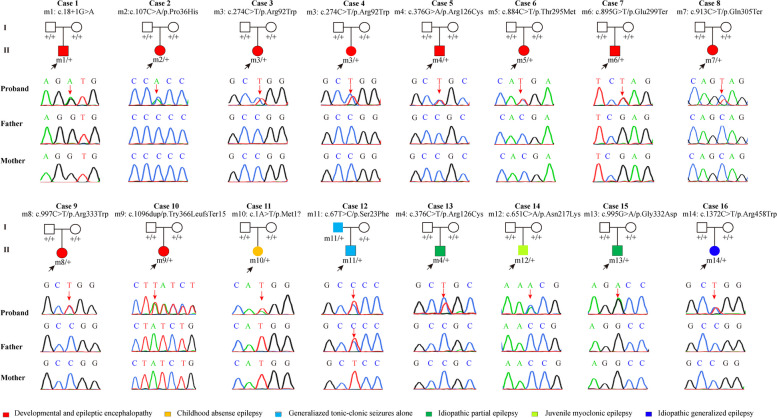
Genetic data of cases with *SLC2A1* variants. Pedigrees and DNA sequencing chromatograms of the 16 cases with *SLC2A1* variants and their corresponding phenotypes. Individuals with variants were indicated by m/ + , and those without variants were indicated by + / +

A total of 14 heterozygous *SLC2A1* variants were identified in 16 families, including 13 de novo and one co-segregation variants. Among these variants, nine have been reported previously, including seven missense (c.67T > C/p.Ser23Phe, c.107C > A/p.Pro36His, c.274C > T/p.Arg92Trp, c.376C > T/p.Arg126Cys, c.884C > T/p.Thr295Met, c.997C > T/p.Arg333Trp, and c.1372C > T/p.Arg458Trp), one splicing (c.18 + 1G > A), and one start-lost (c.1A > T/p.Met1?) variants. The remaining five variants were novel, including two missense (c.651C > A/p.Asn217Lys and c.995G > A/p.Gly332Asp), two nonsense (c.895G > T/p.Glu299Ter and c.913C > T/p.Gln305Ter), and one frameshift (c.1096dup/p.Tyr366LeufsTer15) variants (Fig. 1 and Table 1).

**Table 1:** Clinical features of the individuals with *SLC2A1* variants

Case No	Variants (NM_006516)	Source	Sex/ Age	Seizure onset age	Seizure type/frequency	EEG	Brain MRI	Development	Diagnosis
1	c.18 + 1G > A	de novo	M/ 6 years	2 years	CPS (1–3 times/month)	Bilateral parietal, occipital, and posterior temporal slow and sharp-slow waves	Normal	DD	DEE
2	c.107C > A/ p.Pro36His	de novo	F/ 1years	4 months	CPS (2–3 times/week); FS and SE (once)	Bilateral frontal and central spike waves	Normal	DD	DEE
3	c.274C > T/ p.Arg92Trp	de novo	F/ 13 years	1 years	CPS (10–50 times/day); GTCS (1–2 times/year before age 5)	Diffused spike-slow waves, predominately in frontal and temporal region	Normal	DD, ADHD, ID	DEE
4	c.274C > T/ p.Arg92Trp	de novo	F/ 23 years	2 years	GTCS (4 times/month)	Bilateral frontal–temporal and central-parietal-temporal spike-slow waves	Normal	ID	DEE
5	c.376C > T/ p.Arg126Cys	de novo	M/ 9 years 9 months	4 years	CPS; FS (once)	3–4 Hz slow waves	Normal	DD, ID	DEE
6	c.884C > T/ p.Thr295Met	de novo	F/ 10 years	7 months	GTCS (3 times for 7 years); FS (once)	Bilateral frontal, temporal, and central 2.5–3.5 Hz spike-slow and slow waves	NA	DD, ADHD	DEE
7	c.895G > T/ p.Glu299Ter	de novo	M/ 6 years 5 months	2 years	FS (2–6 times/year); atonic (2–6 times/year); CPS (1–3 times/day)	Diffused 2.5–3.5 Hz slow and spike-slow waves	Normal	DD	DEE
8	c.913C > T/ p.Gln305Ter	de novo	F/ 14 years	6 months	CPS (4–5 times/day); GTCS (once)	Right frontal-central and left central-parietal-temporal sharp and sharp-slow waves	Malacia focus after corpus callosum disconnection surgery	Mild ID, DD	DEE
9	c.997C > T/ p.Arg333Trp	de novo	F/ 3 years 5 months	1 year 6 months	MAS (> 10 times/day); GTCS (1–3 times/month)	Polyspike-slow wave and bilateral central, parietal spike-slow waves	NA	DD	DEE
10	c.1096dup/ p.Tyr366LeufsTer15	de novo	F/ 7 years 3 months	2 years 6 months	Atonic (1 time/2–3 days)	Diffused spike-slow waves	Normal	DD	DEE
11	c.1A > T/ p.Met1?	de novo	F/ 11 years	5 years	AS and MS (> 1 times/day)	Ictal: 3 Hz spike-slow waves; Inter-ictal: left posterior spike and spike-slow waves	Normal	Normal	CAE
12	c.67T > C/ p.Ser23Phe	Paternal	M/ 6 years 3 months	6 years 2 months	GTCS (2 times in 1 month)	Normal	Normal	Normal	GTCA
12–1	c.67T > C/ p.Ser23Phe	NA	M/ NA	10 years	GTCS	Diffused spike-slow waves	NA	Normal	GTCA
13	c.376C > T/ p.Arg126Cys	de novo	M/ 15 years	13 years	CPS (1–2 times/day); GTCS (1 time/2day for 4 months)	Right frontal-central and bilateral parietal-occipital spike-slow waves; diffused 3–3.2 Hz spikes-slow wave	NA	Normal	IPE
14	c.651C > A/ p.Asn217Lys	de novo	M/ 18 years	14 years	GTCS (once); MS (1–2 times/month)	Diffused spike-slow waves	Normal	Normal	JME
15	c.995G > A/ p.Gly332Asp	de novo	M/ 8 years 5 months	4 years	CPS (1 time/2–3 months); GTCS (1 time/year)	Bilateral frontal, temporal, and occipital spike and spike-slow waves	Normal	Normal	IPE
16	c.1372C > T/ p.Arg458Trp	de novo	F/ 26 years	13 years	GTCS (7 times for 13 years); AS (1 time/1–2 months)	3–4 Hz spike-and-slow waves	Normal	Normal	IGE

*Abbreviations*: *ADHD* Attention deficit hyperactivity disorder, *AS* Absence seizures, *CAE* Childhood absence epilepsy, *CPS* Complex partial seizures, *DD* Developmental delay, *DEE* Developmental and epileptic encephalopathy, *F* Female, *FS* Febrile seizures, *GTCA* Generalized tonic-clonic seizures alone, *GTCS* Generalized tonic-clonic seizures, *ID* Intellectual disability, *IGE* Idiopathic generalized epilepsy, *IPE* Idiopathic partial epilepsy, *JME* Juvenile myoclonic epilepsy, *M* Male, *MAE* Myoclonic-atonic epilepsy, *MAS* Myoclonic-atonic seizure, *MS* Myoclonic seizure, *NA* Not available

All the *SLC2A1* variants in this study were absent in the gnomAD-all populations. The missense variants were predicted to be “damaging” by at least 11 in silico algorithms. According to the ACMG guidelines, three missense variants (c.67T > C/p.Ser23Phe, c.651C > A/p.Asn217Lys, and c.995G > A/p.Gly332Asp) were evaluated as “likely pathogenic” and the remaining 11 variants were rated as “pathogenic” (Table 2).

**Table 2:** Genetic characteristics and ACMG scorings of *SLC2A1* variants

Variant (NM_006516)	Inheritance	MAF (gnomAD-all population)	in silico prediction^a^	ACMG (scoring)
c.18 + 1G > A	de novo	0	/	P (PVS1 + PS1 + PS2 + PM2)
c.107C > A/p.Pro36His	de novo	0	22	P (PS1 + PS2 + PM2 + PP3)
c.274C > T/ p.Arg92Trp	de novo	0	22	P (PS1 + PS2 + PM2 + PP3)
c.376C > T/p.Arg126Cys	de novo	0	23	P (PS1 + PS2 + PM2 + PP3)
c.884C > T/p.Thr295Met	de novo	0	23	P (PS1 + PS2 + PM2 + PP3)
c.895G > T/p.Glu299Ter	de novo	0	12	P (PVS1 + PS2 + PM2 + PP3)
c.913C > T/p.Gln305Ter	de novo	0	12	P (PVS1 + PS2 + PM2 + PP3)
c.997C > T/p.Arg333Trp	de novo	0	23	P (PS1 + PS2 + PM2 + PP3)
c.1096dup/p.Tyr366LeufsTer15	de novo	0	/	P (PVS1 + PS2 + PM2)
c.1A > T/p.Met1?	de novo	0	9	P (PVS1 + PS1 + PS2 + PM2 + PP3)
c.67T > C/p.Ser23Phe	Paternal	0	16	LP (PS1 + PM2 + PP1 + PP3)
c.651C > A/p.Asn217Lys	de novo	0	11	LP (PS2 + PM2 + PP3)
c.995G > A/p.Gly332Asp	de novo	0	22	LP (PS2 + PM2 + PP3)
c.1372C > T/p.Arg458Trp	de novo	0	22	P (PS1 + PS2 + PM2 + PP3)

*Abbreviations*: *ACMG* American College of Medical Genetics and Genomics, *LP* Likely pathogenic, *P* Pathogenic, *MAF* Minor allele frequency, *PS1* Same amino acid change as a previously established pathogenic variant regardless of nucleotide change, *PS2 *de novo in a patient with the disease and no family history, *PM2* Absent in population databases, *PP3* Multiple lines of computational evidence support a deleterious effect on the gene/gene product, *PVS1* Null variant (nonsense, frameshift, canonical + / − 1 or 2 splice sites, initiation codon, single or multi-exon deletion) in a gene where loss of function (LOF) is a known mechanism of disease

^a^Variant predicted to be deleterious out of 23 prediction tools according to VarCards (www.genemed.tech/varcards/)

None of the 16 cases had pathogenic or likely pathogenic variants in other genes known to be associated with epileptic phenotypes [12].

### Clinical features of the cases with *SLC2A1* variants

In this study, *SLC2A1* variants were identified in 16 epilepsy cases. The detailed clinical manifestations of the patients with *SLC2A1* variants are summarized in Table 1. Brain MRI of one patient (case 8) showed tissue necrosis after callosal transection and the others were normal.

Ten cases (cases 1–10) were diagnosed with developmental and epileptic encephalopathy (DEE). They presented with early-onset seizures, with 8 cases (80.0%) having an onset age of < 2 years. Four of the cases showed frequent seizures (daily). Nine of the patients had developmental delay, four displayed intellectual disability, and two exhibited attention deficit hyperactivity disorder.

The other six cases (cases 11–16) showed juvenile/childhood-onset seizures and all showed normal development. Four cases mainly exhibited generalized seizures, including absence, myoclonic, and generalized tonic-clonic seizures. Two cases presented with generalized tonic-clonic seizures and complex partial seizures. The diagnoses of the six cases were idiopathic partial epilepsy in two cases, as well as juvenile myoclonic epilepsy, childhood absence epilepsy, epilepsy with generalized tonic-clonic seizures alone, and idiopathic generalized epilepsy in the other four each.

The proband of case 12 experienced two generalized tonic-clonic seizures within one month. He received early genetic diagnosis at the age of 6 years 3 months and showed normal development. All other cases received delayed diagnosis, with the longest delay being 22 years.

### Molecular subregional effects of *SLC2A1*

**Fig. 2:**
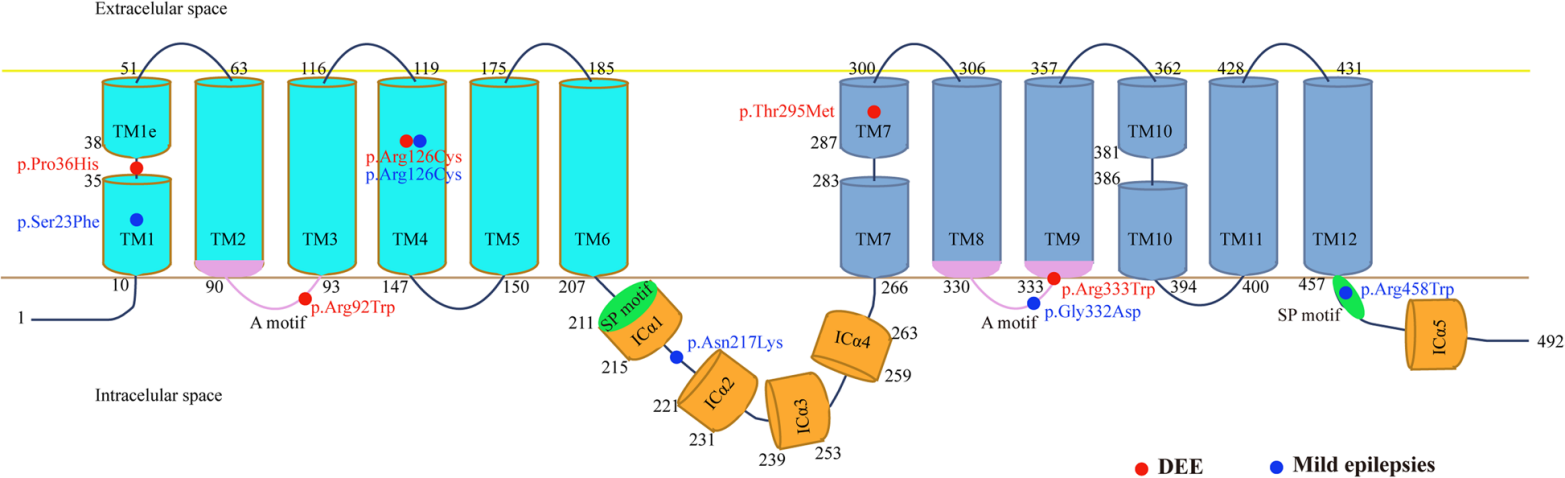
Schematic diagram of the GLUT1 protein and the localization of the *SLC2A1* missense variants identified in this study. Variants identified in patients with developmental and epileptic encephalopathy are shown in red and those with mild epilepsies are shown in blue. The two proper signature motifs of the major facilitator superfamily (MFS) in GLUT1 protein were called A motifs. The two signature motifs of the sugar transporter subfamily were called SP motifs. TM, transmembrane segment

The GLUT1 protein contains an amino-terminal (NT) domain and a carboxy-terminal (CT) domain. There are 12 transmembrane segments in total, with the first six (TM1-TM6) forming the NT domain and the last six (TM7-TM12) forming the CT domain. The NT and CT domains are connected by an intracellular helical bundle (IC) domain comprising four short α-helices (Icα1–Icα4 domains) [13]. Previous studies have suggested that the phenotypic severity is associated with the sub-molecular effects of genetic variants [14, 15]. We therefore explored the location of missense variants identified in this study and their associations with phenotype (Fig. 2). We found that the DEE-associated variants tended to cluster in the transmembrane region, whereas the mild epilepsy-associated variants tended to be located in the regions outside the transmembrane region, such as the IC domain and the CT intracellular region, suggesting a potential molecular subregional effect.

### Temporal expression of *SLC2A1*

**Fig. 3:**
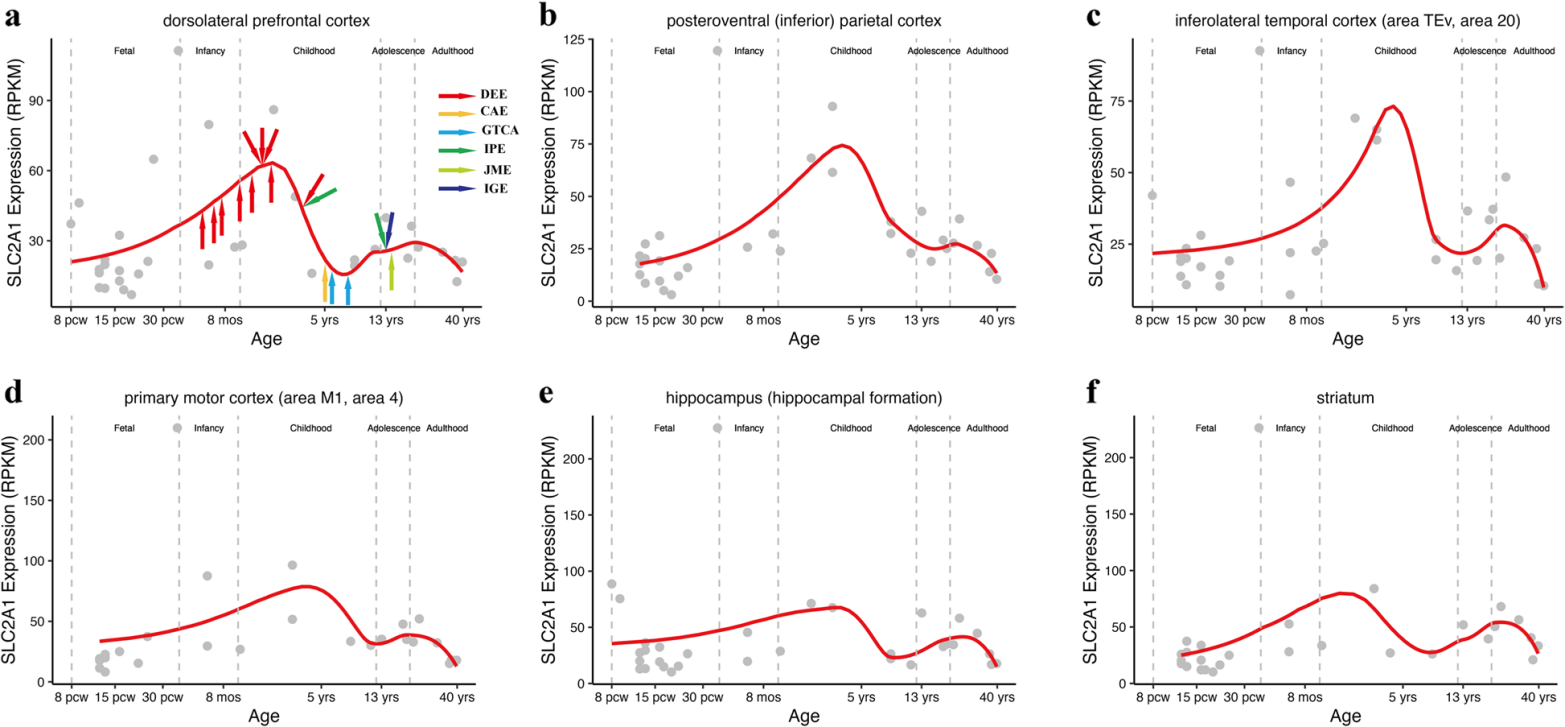
The temporal expression pattern of *SLC2A1* in the human brain in different brain areas that was retrieved from the BrainSpan database. The onset age of the 17 individuals were illustrated in (**a**). CAE, childhood absence epilepsy; DEE, developmental and epileptic encephalopathy; GTCA: generalized tonic-clonic seizures alone; IGE, idiopathic generalized epilepsy; IPE, idiopathic partial epilepsy; JME: juvenile myoclonic epilepsy

In this study, the 17 patients with *SLC2A1* variants displayed a broad range of seizure onset, from 4 months to 14 years, with a median onset age of 2 years 6 months (Fig. 3). Our recent studies have suggested a correlation of the genetic-dependent (expression) stage with the onset age and the outcomes of genetic diseases [16–18]. We then analyzed the temporal expression pattern of *SLC2A1* in the human brain. *SLC2A1* is expressed at relatively high level throughout the whole life span, from embryonic to adult stages with two peaks at approximately four and 14 years (Fig. 3). The wide range of onset age was generally consistent with the temporal expression of *SLC2A1*.

The patients with DEE in this study had onset age during infancy and early childhood. The variants in these patients were clustered in the transmembrane region that is potentially associated with severe damage. In addition, five of the six cases with mild epilepsy had seizures at late childhood or early adolescence. Their variants tended to be located at regions outside the transmembrane that are potentially associated with mild damage. The findings suggested that the onset age correlated with the damaging effects of the variants (Fig. 3a).

## Discussion

In this study, 14 *SLC2A1* heterozygous variants, including 13 de novo and one co-segregation, were identified in 16 epilepsy cases. These variants were absent in the gnomAD-all populations and were predicted to be damaging by multiple in silico tools. They were evaluated to be “pathogenic” or “likely pathogenic” according to the ACMG guidelines. Further analysis suggested that *SLC2A1* is expressed during the embryonic and late childhood stages, explaining the broad onset age of the patients.

Previous studies estimated that about 90% patients with *SLC2A1* variants presented with classical GLUT1DS [6, 19]. The onset age of seizures is typically before the age of two years [7]. Cognitive impairment, ranging from learning disabilities to severe intellectual disability, is typical in GLUT1DS [19]. Patients also showed acquired microcephaly and complex movement disorders [20, 21]. In the present study, 10 of the 16 cases showed early-onset seizures and were diagnosed with DEE, while the remaining 6 cases (37.5%) presented with less severe phenotypic manifestations with late-onset seizures and normal development. Under the pressure of natural selection, genetic variants with less damaging effects would be more common than those with more severe damaging effects, subsequently leading to mild and common diseases. Therefore, clinically more attention should be paid to mild epilepsies caused by *SLC2A1* variants.

The GULT1 protein has 12 transmembrane segments, with the first six (TM1–TM6) forming the NT domain and the last six (TM7–TM12) forming the CT domain [22, 23]. The CT domain is essential for glucose binding and the NT domain undergoes rotation to allow the passage of glucose [5]. Additionally, the contact between TM1 and TM7 on the extracellular side serves as the primary constituent of the extracellular gate [5, 13]. The IC domain of the GULT1 protein may serve as a latch that ensures the closure of the intracellular gate when it adopts an outward-facing conformation [13]. This study showed that DEE-associated variants tend to cluster in the transmembrane region, whereas the mild epilepsy-associated variants tend to be located at the regions outside the transmembrane, suggesting a potential molecular subregional effect.

Our recent studies have suggested a correlation of the genetic-dependent (expression) stage with the onset age and the outcomes of genetic diseases [16–18]. The temporal expression of *SLC2A1*, which is expressed throughout the embryonic and adult stages, with two peaks at approximately four and 14 years, is generally consistent with the seizure onset age. In this study, 9 of the 10 patients with DEE showed early-onset age. They had variants that tended to cluster in the transmembrane region, which were potentially associated with severe damage, suggesting a potential correlation between the age of disease onset and the damaging effects of the variants.

Ketogenic diet (KD) is a high-fat and low-carbohydrate diet that plays a vital role in the treatment of GLUT1DS [6, 24]. Early diagnosis and early treatment with KD are associated with improved neurologic outcomes of GLUT1DS [5, 25, 26]. In this study, early and accurate genetic diagnosis was only made in one patient who showed normal development. Other cases, particularly those patients with DEE, received delayed diagnosis. Early genetic testing is necessary for the early genetic diagnosis for patients with *SLC2A1* variants.

## Conclusions

*SLC2A1* variants are associated with late-onset mild epilepsy, which is consistent with the genetic-dependent stage of *SLC2A1*. Early genetic diagnosis implies the significance of treatment in patients with *SLC2A1* variants.

## Data Availability

The data supporting the findings of this study are available from the corresponding author upon reasonable request.

## Abbreviations

ACMG: American College of Medical Genetics and Genomics
CT: Carboxy-terminal
DEE: Developmental and epileptic encephalopathy
GLUT1: Glucose transporter type 1
GLUT1DS: Glucose transporter type 1 deficiency syndrome
IC: Intracellular helical bundle
KD: Ketogenic diet
MAF: Minor allele frequency
MRI: Magnetic resonance imaging
NT: Amino-terminal
WES: Whole-exome sequencing
